# Supplementary material for: Barriers to Compliance with National Guidelines Among Children Hospitalized with Community-Acquired Pneumonia in Vietnam and the Implications
Source: Antibiotics (Basel). 2025 Jul 15;14(7):709. doi: 10.3390/antibiotics14070709 (PMC12291924; doi:10.3390/antibiotics14070709)
Supplement: Supplementary file 1 [file antibiotics-14-00709-s001.zip › antibiotics-3706510-supplementary.pdf]

Supplementary Tables

Table S1. Summary of findings regarding antibiotic use in the treatment of Community-Acquired Pneumonia in Children in Vietnam

Location and Study Period	Sample Size	Patient Characteristics	Initial Antibiotic Regimen Characteristics	Antibiotics Used Initially and Appropriateness
Bach Mai Hospital – 2018 [109]	193 children with CAP, aged 2 months to 5 years	• Prior antibiotic use*: 52.3%<br>• Co-morbidities: 12.4%<br>**Classified [110]**:<br>• P: 49.7%<br>• SP: 47.7%<br>• VSP: 1.6%	**P Patients:**<br>• Single regimen: 94.8%<br>• Combined regimen: 5.2%<br>**SP Patients:**<br>• Single regimen: 79.3%<br>• Combined regimen: 20.7%<br>**VSP Patients:**<br>• Single regimen: 80.0%<br>• Combined regimen: 20.0%	• Penicillin ± β-lactamase inhibitor: 66.3%<br>• C2G: 0.4%<br>• C3G: 23.7%<br>• Macrolide: 6.2%<br>• Aminoglycoside: 9.8%<br>• Glycopeptide: 0.8%
National Children's Hospital, 2017 [111]	209 children with CAP, aged 2 months to 5 years	• Prior antibiotic use*: 29.67%<br>• Co-morbidities: 10.05%<br>**Classified [80]:**<br>• P: 47.85%<br>• SP: 47.37%<br>• VSP: 4.78%	Mostly single antibiotic regimen: 78.74%	Penicillin ± β-lactamase inhibitor: 52.15%<br><br>Duration treatment antibiotics: 6.0 (4.0–8.0) days
Nghe An Obstetrics and Pediatrics Hospital in 2021 [40]	90 children with CAP, aged 2 months to 5 years	• Prior antibiotic use*: 51.1%.<br>• Co-morbidities: N/A<br>**Classified [80]:**<br>• P: 17.8%,<br>• SP: 82.2%,	• Single regimen: 90.0%<br>• Combined regimen: 10.0%	• Penicillin/β-lactamase inhibitor: 17.2%<br>• C3G: 52.5%<br>• Macrolide: 27.3%<br>• Aminoglycoside: 3.0%
Phu Tho General Hospital – 2021 [112]	159 children with CAP, aged 2 months to 5 years	• Prior antibiotic use*: 85.12%.<br>• Co-morbidities: 35.46%.<br>**Classified per [80]:**<br>• P: 96.5%,<br>• SP: 3.6%.<br>•	**P Patients:**<br>• Single regimen: 87.5%<br>• Combined regimen: 12.5%.<br>**SP Patients**:<br>• Single regimen: 80.0%<br>• Combined regimen: 20.0%.	• Penicillin ± β-lactamase inhibitor: 0%,<br>• C3G: 63.7%,<br>• C4G: 10.6%, Carbapenem: 0.4%<br>• Macrolide: 1.3%<br>• Aminoglycoside: 23.9%.
Quang Nam Central General Hospital, 2024 [41]	170 children with CAP, aged 2 months to 5 years	• Prior antibiotic use*: N/A<br>• Co-morbidities: 84.7%<br>**Classified [80]:**<br>• P: 87.6%,<br>• SP: 12.4%,	**P Patients:**<br>• Single regimen: 57.7%<br>• Combined regimen: 42.3%<br>**SP Patients:**<br>• Single regimen: 61.9%<br>• Combined regimen: 38.1%	• Penicillin/β-lactamase inhibitor: 8.8%<br>• C3G: 41.2%<br>• C3G+Aminoside: 17.6%<br>• Penicillin/β-lactamase + aminoglycoside: 11.8%<br>• Others: 20.6%<br><br>• Dosage of antibiotics appropriate: 49.8%<br>• Duration treatment antibiotics: 6.4±2.1 days
Can Tho Children's Hospital, 2024 [42]	301 children with CAP, 86.4% aged under 5 years	• Prior antibiotic use*: N/A<br>• Co-morbidities: N/A<br>**Classified [80]:**<br>• P: 88.0%,	• Single regimen: 66.4%<br>• Combined regimen: 33.6%	• Penicillin/β-lactamase inhibitor: 32.9%<br>• C2G: 23,9%<br>• C3G: 57.8%<br>• Macrolide: 15.9%

		• SP: 12.0%,		• Aminoglycoside: 7.0%<br>• Carbapenem: 1.7%<br>• Glycopeptide: 1.3%<br><br>• Dosage of antibiotics appropriate: 87.7%<br>• Duration treatment antibiotics: 6-10 days (63.5%)
**Location and Study Period**	**Sample Size**	**Patient Characteristics**	**Initial Antibiotic Regimen Characteristics**	**Antibiotics Used Initially and Appropriateness**
Bach Mai Hospital – 2018 [110]	193 children with CAP, aged 2 months to 5 years	• Prior antibiotic use*: 52.3%<br>• Co-morbidities: 12.4%<br>**Classified [111]**:<br>• P: 49.7%<br>• SP: 47.7%<br>• VSP: 1.6%	**P Patients:**<br>• Single regimen: 94.8%<br>• Combined regimen: 5.2%<br>**SP Patients:**<br>• Single regimen: 79.3%<br>• Combined regimen: 20.7%<br>**VSP Patients:**<br>• Single regimen: 80.0%<br>• Combined regimen: 20.0%	• Penicillin ± β-lactamase inhibitor: 66.3%<br>• C2G: 0.4%<br>• C3G: 23.7%<br>• Macrolide: 6.2%<br>• Aminoglycoside: 9.8%<br>• Glycopeptide: 0.8%
National Children's Hospital, 2017 [112]	209 children with CAP, aged 2 months to 5 years	• Prior antibiotic use*: 29.67%<br>• Co-morbidities: 10.05%<br>**Classified [80]:**<br>• P: 47.85%<br>• SP: 47.37%<br>• VSP: 4.78%	Mostly single antibiotic regimen: 78.74%	Penicillin ± β-lactamase inhibitor: 52.15%<br><br>Duration treatment antibiotics: 6.0 (4.0–8.0) days
Nghe An Obstetrics and Pediatrics Hospital in 2021 [40]	90 children with CAP, aged 2 months to 5 years	• Prior antibiotic use*: 51.1%.<br>• Co-morbidities: N/A<br>**Classified [80]:**<br>• P: 17.8%,<br>• SP: 82.2%,	• Single regimen: 90.0%<br>• Combined regimen: 10.0%	• Penicillin/β-lactamase inhibitor: 17.2%<br>• C3G: 52.5%<br>• Macrolide: 27.3%<br>• Aminoglycoside: 3.0%
Phu Tho General Hospital – 2021 [113]	159 children with CAP, aged 2 months to 5 years	• Prior antibiotic use*: 85.12%.<br>• Co-morbidities: 35.46%.<br>**Classified per [80]:**<br>• P: 96.5%,<br>• SP: 3.6%.<br>•	**P Patients:**<br>• Single regimen: 87.5%<br>• Combined regimen: 12.5%.<br>**SP Patients**:<br>• Single regimen: 80.0%<br>• Combined regimen: 20.0%.	• Penicillin ± β-lactamase inhibitor: 0%,<br>• C3G: 63.7%,<br>• C4G: 10.6%, Carbapenem: 0.4%<br>• Macrolide: 1.3%<br>• Aminoglycoside: 23.9%.
Quang Nam Central General Hospital, 2024 [41]	170 children with CAP, aged 2 months to 5 years	• Prior antibiotic use*: N/A<br>• Co-morbidities: 84.7%<br>**Classified [80]:**<br>• P: 87.6%,<br>• SP: 12.4%,	**P Patients:**<br>• Single regimen: 57.7%<br>• Combined regimen: 42.3%<br>**SP Patients:**<br>• Single regimen: 61.9%<br>• Combined regimen: 38.1%	• Penicillin/β-lactamase inhibitor: 8.8%<br>• C3G: 41.2%<br>• C3G+Aminoside: 17.6%<br>• Penicillin/β-lactamase + aminoglycoside: 11.8%<br>• Others: 20.6%<br><br>• Dosage of antibiotics appropriate: 49.8%<br>• Duration treatment antibiotics: 6.4±2.1 days
Can Tho	301 children with CAP,	• Prior antibiotic use*: N/A	• Single regimen: 66.4%<br>• Combined regimen: 33.6%	• Penicillin/β-lactamase inhibitor: 32.9%

Children's Hospital, 2024 [42]	86.4% aged under 5 years	• Co-morbidities: N/A<br>**Classified [80]:**<br>• P: 88.0%,<br>• SP: 12.0%,		• C2G: 23,9%<br>• C3G: 57.8%<br>• Macrolide: 15.9%<br>• Aminoglycoside: 7.0%<br>• Carbapenem: 1.7%<br>• Glycopeptide: 1.3%<br>• Dosage of antibiotics appropriate: 87.7%<br>• Duration treatment antibiotics: 6-10 days (63.5%)

NB: C2G: second-generation cephalosporins; C3G: third-generation cephalosporins; P: Pneumonia; SP: Severe Pneumonia; VSP: Very Severe Pneumonia. Regimen: Treatment regimen; Co-morbidities: Coexisting conditions. Please refer to the published paper for details of the references

Table S2 Ethical Approval

MINISTRY OF HEALTH	**SOCIALIST REPUBLIC OF VIETNAM**
**E HOSPITAL**	**Independence – Freedom – Happiness**
**No: 159/PCT-HDDD**	-------------------------

Hanoi, June 23, 2023

ETHICS COMMITTEE APPROVAL FOR
BIOMEDICAL RESEARCH

Pursuant to Decision 725/QD-BVE dated March 29, 2023 on the establishment of the Council and Secretariat of the Ethics Council in Biomedical Research at the Hospital E for the 2023-2027 term;

Based on the minutes of the research proposal evaluation by the project leader: Dr. Vu Thi Thu Huong;

The E Hospital Biomedical Research Ethics Council approves the ethical and scientific aspects of research for the scientific research topic:

1. Topic name: **Analysis of the current status of antibiotic use in the treatment of community-acquired pneumonia in children from 2 months to 5 years old and some related factors at the Central E Hospital**
2. Project leader: Dr. Vu Thi Thu Huong
3. Host agency: E Hospital
4. Research location: E Hospital

Require research participants to fully comply with biomedical research regulations.

CHAIRMAN OF THE BOARD

(Signed)

Prof. Dr. Le Ngoc Thanh

BỆNH VIỆN E
HỘI ĐỒNG ĐẠO ĐỨC TRONG NGHIÊN CỨU Y SINH HỌC
Số: 159/PCT - HĐĐĐ

CỘNG HÒA XÃ HỘI CHỦ NGHĨA VIỆT NAM
Độc lập – Tự do – Hạnh phúc

Hà Nội, ngày 23 tháng 6 năm 2023

PHIẾU CHẤP NHẬN CỦA HỘI ĐỒNG ĐẠO ĐỨC TRONG NGHIÊN CỨU Y SINH HỌC

Căn cứ Quyết định 725/QĐ-BVE ngày 29 tháng 3 năm 2023 về việc thành lập Hội đồng, Ban cố vấn, Ban thư ký của Hội đồng đạo đức trong nghiên cứu Y sinh học cấp cơ sở tại Bệnh viện E nhiệm kỳ 2023 – 2027;

Căn cứ biên bản đánh giá đề cương nghiên cứu và phiếu đánh giá dành cho thành viên Hội đồng đạo đức ngày 19 tháng 6 năm 2023;

Hội đồng đạo đức trong nghiên cứu Y sinh học Bệnh viện E chấp thuận về các khía cạnh đạo đức và tính khoa học trong nghiên cứu cho đề tài nghiên cứu khoa học:

1. Tên đề tài: **Phân tích thực trạng sử dụng kháng sinh trong điều trị viêm phổi mắc phải tại cộng đồng ở trẻ em từ 2 tháng đến 5 tuổi và một số yếu tố liên quan tại Bệnh viện E Trung ương**
2. Chủ nhiệm đề tài: TS Vũ Thị Thu Hương
3. Cơ quan chủ trì: Bệnh viện E
4. Địa điểm nghiên cứu: Bệnh viện E

Yêu cầu các bên tham gia nghiên cứu thực hiện đầy đủ các quy định về nghiên cứu y sinh học.

CHỦ TỊCH HỘI ĐỒNG

GS. TS. Lê Ngọc Thành
